# Whole transcriptome data of primary human NK cells under hypoxia and interleukin 15 priming: A 2×2 factorial design experiment

**DOI:** 10.1016/j.dib.2017.07.018

**Published:** 2017-07-15

**Authors:** Ana Sofia Figueiredo, Doreen Killian, Jutta Schulte, Carsten Sticht, Holger A. Lindner

**Affiliations:** a Department of Anesthesiology and Surgical Intensive Care Medicine, University Medical Center Mannheim, Medical Faculty Mannheim, Heidelberg University, Germany; b Medical Research Center, University Medical Center Mannheim, Medical Faculty Mannheim, Heidelberg University, Germany

**Keywords:** Microarray, Hypoxia, Interleukin 15, Natural Killer cells

## Abstract

Natural Killer (NK) cells mediate innate immunity against cancer and intracellular infection, at that, operating in often oxygen-deprived environments. We performed a microarray experiment with a 2×2 factorial design to profile gene expression in human NK cells (Velasquez et al., 2016) [1]. In this experiment, NK cells from 5 healthy volunteers were primed or not for 6 h with the survival factor and inflammatory cytokine interleukin 15 (IL-15) under hypoxic or normoxic culture conditions (20 samples in total). Here, we provide details on the culture setup that govern the actual O_2_ partial pressure (pO_2_) experienced by the cells, as well as on the RNA extraction procedure used, which we optimized from commercial spin column protocols to obtain highly concentrated total RNA. We present a quality control analysis of the normalized microarray data, as well as overviews for differentially regulated genes. These data provide insights into NK cell transcriptional responses to immune stimulation under physiologically relevant low oxygen conditions. This dataset is deposited in the Gene Expression Omnibus database (accession number GSE70214).

## Specifications Table

**Table:** 

Subject area	*Medicine, Biology*
More specific subject area	*Immunology, Hypoxia*
Type of data	*Transcriptome data*
How data was acquired	*For transcriptome profiling, we used Hugene-2_0-st-type arrays (Affymetrix) on an Affymetrix GeneChip platform, and a Custom CDF Version 17 with Entrez-based gene definitions for array annotation.*
Data format	*Raw, normalized, analyzed*
Experimental factors	*Total RNA was extracted from human NK cells cultured under hypoxia or normoxia following a 6 h-period of IL-15 priming or resting.*
Experimental features	*Suspension cultures of isolated NK cells in gas-permeable plates were placed in a standard or oxygen-controlled CO*_*2*_*incubator set to 1% O*_*2*_*. We used the mirVana buffer system combined with Pure Link micro kit spin cartridges for RNA extraction from RNAlater stored cells. Microarray data were normalized using quantile normalization and robust multiarray average (rma) background correction. We performed one-way analysis of variance to identify differentially expressed genes.*
Data source location	*Mannheim, Germany*
Data accessibility	*Raw and normalized microarray data are deposited in the Gene Expression Omnibus database (*www.ncbi.nlm.nih.gov/geo/*; accession no.*GSE70214[1]).

## Value of the data

• This data set provides information on transcriptome level changes in human NK cells through IL-15 priming under hypoxia and normoxia [1].

• Described experimental features of the cell culture set up for hypoxia and normoxia as well as the RNA extraction protocol will enable the reproduction and comparison of the reported data by others.
• To contextualize this data set, we searched for other data sets of high-throughput functional genomics data in open repositories. Specifically, we queried the GEO data repository [2] with the following keyword combinations (1–3): (1) “NK cells primed hypoxia”, (2) “NK cells IL-15 hypoxia” OR “NK cells hypoxia”, and (3) “NK cells IL-15 primed”. To date, this search returned two relevant hits [3], [4], excluding our own data set.
• Information on the transcriptome profiles supports the reuse of the deposited transcriptome data for comparative analyses of differential gene expression and pathways regulation in primed NK cells under hypoxia.

## 1 Data

**Fig. 1:**
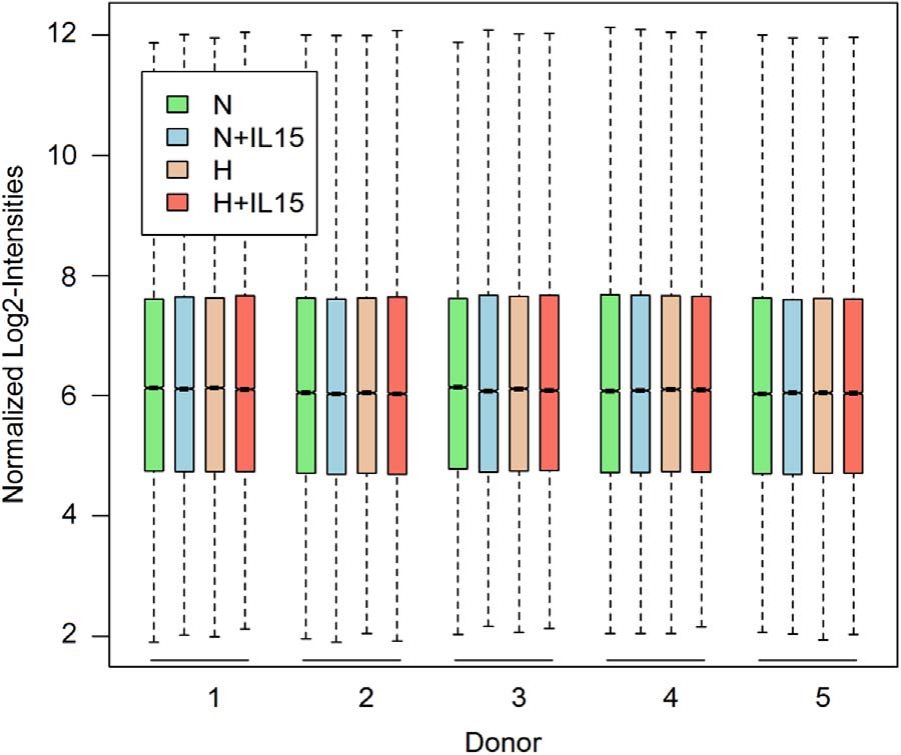
Boxplots for signal intensity distributions of arrays from all donors and experimental conditions after normalization. Experimental conditions are abbreviated as follows: H and N stand for normoxia and hypoxia without priming, respectively, and +IL-15 indicates priming under either condition.

**Fig. 2:**
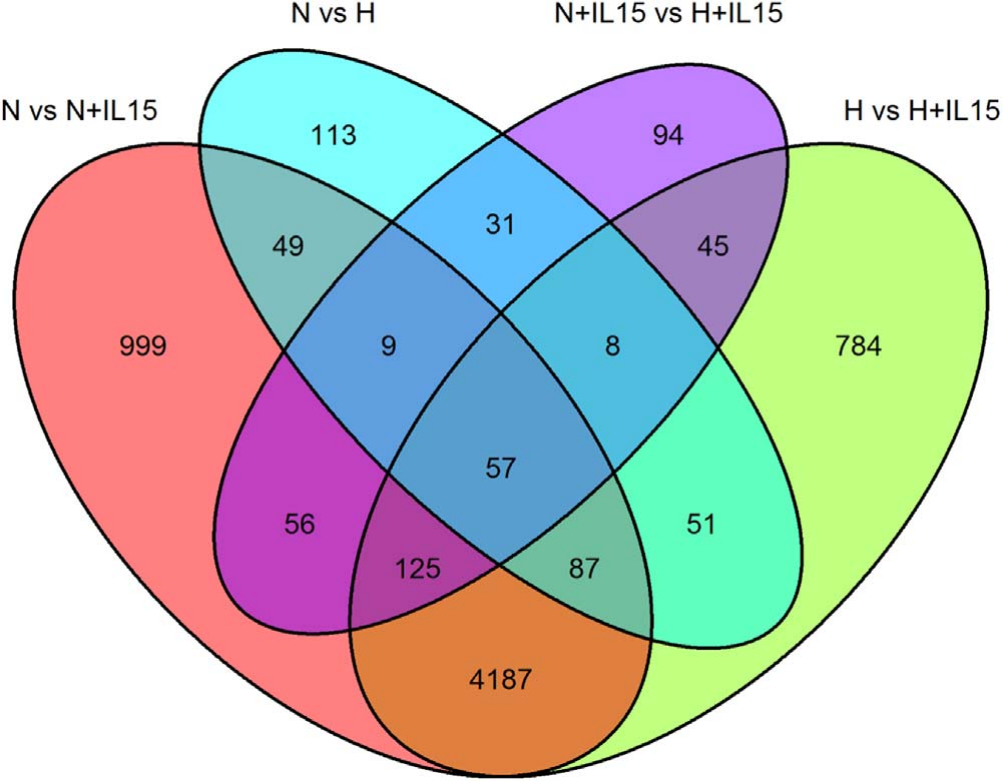
Venn diagram of the significant genes (FDR<0.05) that are differentially expressed for each group. H and N stand for normoxia and hypoxia without priming, respectively, and +IL-15 indicates priming under either condition.

**Fig. 3:**
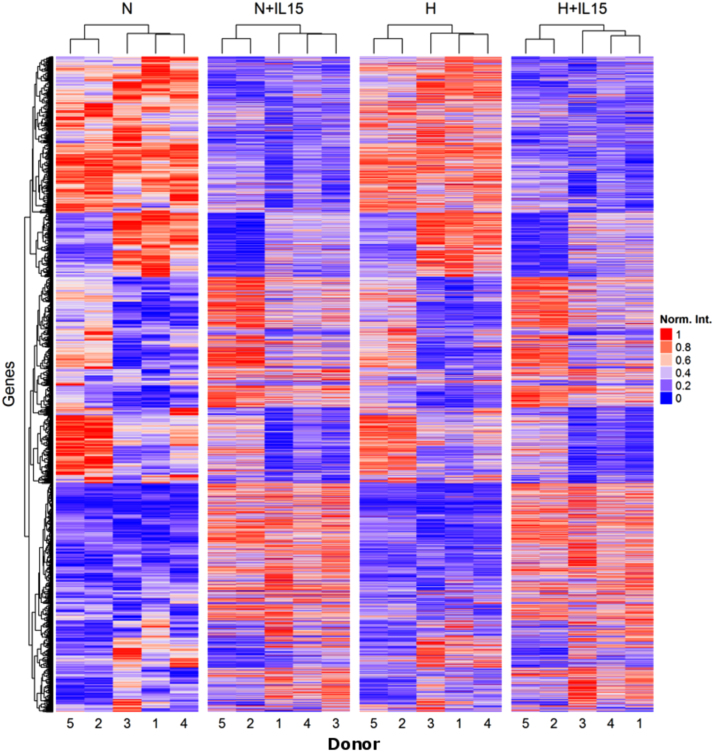
Heatmap of genes significantly regulated by hypoxia and IL-15 priming. H and N stand for normoxia and hypoxia without priming, respectively, and +IL-15 indicates priming under either condition.

**Fig. 4:**
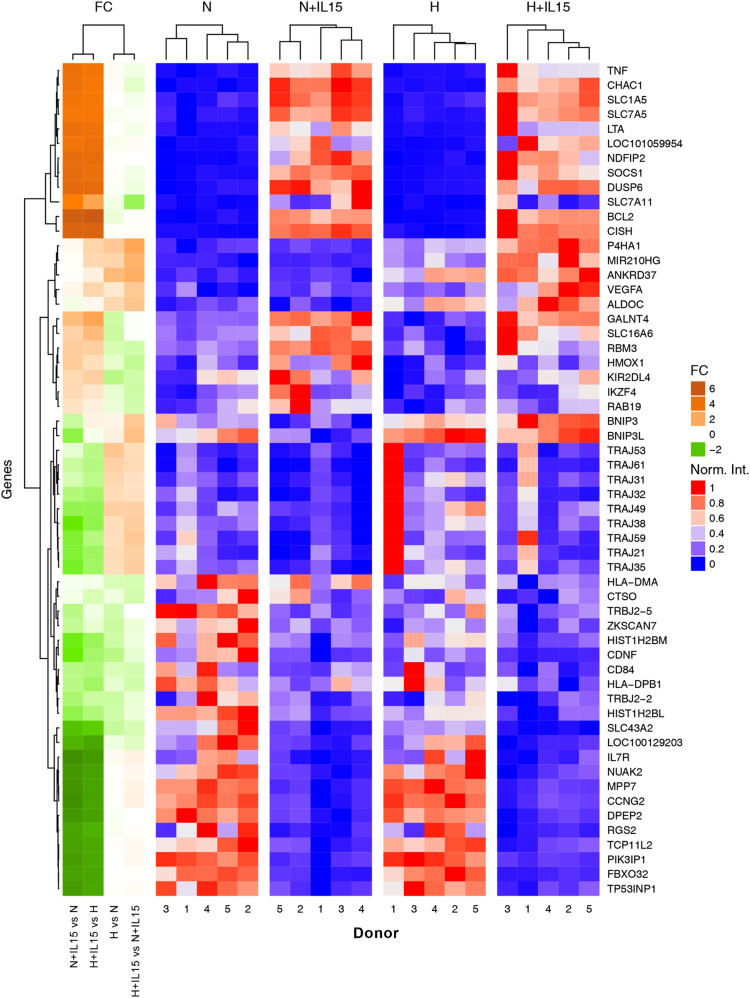
Heatmap of top 10 genes up- or down-regulated by either experimental condition, IL-15 priming (+IL-15) and hypoxia (H) from Fig. 3. N stands for normoxia. Fold changes (FC) for 4 comparisons are displayed as dark orange–green heatmap. Red–blue heatmaps represent normalized intensities. Notably, donor 1 shows high intensities for several TRAJ (T cell receptor alpha joining) genes under hypoxia without IL-15 priming.

Along with the data set, we provide information about the experimental details on the hypoxic cell culture setup that determine the actual pericellular pO_2_, both in a normoxic and hypoxic incubator. Culture medium volume as well as vessel surface area and geometry all influence culture medium depth above the settled cells in an unstirred culture. Because of poor solubility in the liquid phase and continuous cellular consumption, the pO_2_ in the culture medium decreases with depth toward the cells at the bottom of a standard culture dish where its actual value is not known [5]. In addition, we provide a step by step protocol for using the mirVana buffer system in combination with Pure Link micro kit filter columns for extraction of total RNA from cells. We tested the quality of the deposited microarray data for each of the 20 samples (Fig. 1, Supplementary material). Further, we represent the global comparison between the four experimental conditions (Fig. 2, Fig. 3). Fig. 1 depicts the box plots for all microarray experiments after normalization. We illustrate differential gene expression through experimental factors (IL-15 priming and hypoxia) using Venn diagram (Fig. 2) and heatmap representations (Fig. 3, Fig. 4). The differential gene expression data is available as Supplementary material 1.

## 2 Experimental design, materials and methods

### 2.1 Cell culture

Peripheral blood NK cells were isolated, tested for purity, and cultured at 37 °C in a water-saturated atmosphere using an oxygen-controlled (hypoxia) incubator set to 1% O_2_ or a standard CO_2_ incubator as described [1]. Five million cells each were plated in 5 mL volumes into round 5 cm diameter lumox® tissue culture dishes with ultra-thin, gas-permeable film base (Sarstedt, order number: 94.6077.305) to minimize the O_2_ diffusion path. At 16 h, cells were IL-15 primed or not and returned to the respective incubator for another 6 h, as described [1]. At the start of the culture and when returning the cells following priming, it took 1 h for the hypoxia incubator to establish and to reestablish, respectively, a 1% O_2_-atmosphere.

### 2.2 RNA isolation

Cells stored in RNAlater™ Stabilization Solution (Invitrogen) were pelleted for 7 min at 7000g. For isolation of total RNA, reagent solutions were taken from the mirVana™ miRNA Isolation Kit and spin cartridges, wash tubes, and recovery tubes from the PureLink^®^ PCR Micro Kit (Invitrogen). They were applied as follows. The thawed cell pellet was resuspended in 600 µL Lysis/Binding Buffer and mixed with 60 µL miRNA Homogenate Additive by vortexing. During a 10 min-incubation of the mixture on ice, the spin cartridge was centrifuged at 10,000*g* for 1 min, and nuclease-free water was heated to 95 °C. The cell lysate was mixed with 600 µL Acid-Phenol:Chloroform by 30 s of vortexing and centrifuged for 5 min at 10,000*g*. The upper aqueous phase was transferred to a fresh tube and 1.25 volumes of 96% ethanol were added. The lysate/ethanol mixture was loaded onto the spin cartridge on a wash tube followed by a 1 min-centrifugation step at 10,000*g*. The flow through was discarded. Sequential wash steps with 650 µL wash solution 1 and 500 µL each of wash solution 2 and 3 were performed by 1 min-centrifugations at 10,000g followed by an additional 1 min-centrifugation at 14,000*g*. The flow through was discarded after each centrifugation. RNA was eluted into a recovery tube by adding 13 µL of 95 °C nuclease free water, a 1 min-incubation, and 1 min-centrifugation at 14,000*g*. Eluted total RNA was DNAse treated, quantified, and integrity was assessed as described [1]. Samples were stored at −80 °C until use.

### 2.3 Microarray experiment

Hugene-2_0-st-type arrays (Affymetrix) were performed on an Affymetrix GeneChip platform at the Affymetrix Core Facility, Medical Research Center Mannheim (Maria Muciek), and data were preprocessed as described [1].

Here, we used R/Bioconductor to implement the microarray data normalization, quality control and analysis. R is a programming language for data analysis, statistical computing and visualization [6]. Bioconductor is a R-based open source and open development project that enables the analysis of high-throughput data, e.g., microarray data [7].

To control the quality of the microarray samples, we plotted the summary of the signal intensity of each probe array after normalization using the R function Similarities of boxplots between probe arrays is an indicator of good quality experimental procedures (Fig. 1). To evaluate effects of hypoxia and priming, respectively, we selected genes with significant differences in expression through these experimental factors based on their False Discovery Rate (FDR) values as described [1]. Only those with a FDR value<0.05 were considered and are provided as Supplementary material 1 in CSV file format with log2-intensity averages for experimental groups and fold changes. We used the R function from the package VennDiagram [8], to visualize how differential gene expression profiles through each experimental factor overlap (Fig. 2). We implemented a heatmap for these genes (Fig. 3) and subsets of top 10 up- and top 10 downregulated genes (Fig. 4) using the function from the R/Bioconductor package ComplexHeatmap [9]. Intensities are row-normalized between 0 and 1 (Fig. 3, Fig. 4). Fig. 4 additionally includes color-coded fold changes. We used the R/Bioconductor package arrayQualityMetrics [10] to validate the quality of the microarray experiment (Supplementary material 2).
